# Bone-in-bone and sandwich vertebrae in a 6-month-old infant with genetically confirmed fatal osteopetrosis: A case report

**DOI:** 10.1097/MD.0000000000045954

**Published:** 2025-11-21

**Authors:** Fang Hou, Junyan Zhang

**Affiliations:** a Department of Pediatrics, The First People’s Hospital of Datong, Datong, Shanxi Province, China; b Department of Clinical Epidemiology and Evidence-based Medicine, Shanxi Bethune Hospital, Shanxi Academy of Medical Sciences, Tongji Shanxi Hospital, Third Hospital of Shanxi Medical University, Taiyuan, Shanxi Province, China.

**Keywords:** bone-in-bone appearance, osteopetrosis, rare disease, sandwich vertebrae, TCIRG1 gene

## Abstract

**Rationale:**

Autosomal recessive osteopetrosis (ARO) is a subtype of osteopetrosis that is often fatal and typically manifests in infancy with life-threatening complications. This report describes a genetically confirmed case of ARO with characteristic radiographic features and a fatal outcome, highlighting the diagnostic value of X-ray radiographs in early recognition of the disease.

**Patient concerns:**

A 6-month-old female presented with high fever, poor responsiveness, and decreased appetite. Physical examination revealed a bulging anterior fontanel, hepatosplenomegaly (3 cm below the costal margin), and bilateral Babinski signs.

**Diagnoses:**

Laboratory tests showed progressive anemia (hemoglobin 94 g/L), thrombocytopenia (platelet count 52 × 10^9^/L), and elevated C-reactive protein (11.9 mg/L). Images demonstrated thickened cranial bones with features suggestive of increased intracranial pressure. X-rays revealed classical “sandwich vertebrae” and “bone-in-bone” appearances in the spine and long bones. Genetic testing identified compound heterozygous mutations in the *TCIRG1* gene: c.1216G>T:p.D406Y (paternal origin) and c.1384_1386del:p.N462del (maternal origin), confirming the diagnosis of ARO type 1 (OMIM #259700).

**Interventions:**

Supportive and anti-infective treatments were administered. Hematopoietic stem cell transplantation was not performed due to financial constraints.

**Outcomes:**

The patient died of marrow failure and infection.

**Lessons:**

Plain X-ray radiographs have crucial diagnostic value for early identification of ARO in infants. Because computed tomography entails high radiation exposure and is not suitable for infants, X-ray remains the preferred initial imaging modality. Prompt genetic testing and timely hematopoietic stem cell transplantation are essential for improving outcomes in this otherwise fatal disorder.

## 1. Introduction

Osteopetrosis is a rare hereditary skeletal disorder characterized by impaired osteoclast function, leading to increased bone mass and fragility.^[1]^ Osteopetrosis is a group of rare skeletal metabolic disorders characterized by impaired bone resorption, primarily caused by defects in osteoclast function or differentiation.^[1,2]^ Autosomal recessive osteopetrosis (ARO) is a rare subtype of osteopetrosis, with an estimated incidence of approximately 1 in 250,000 live births.^[2]^ ARO typically presents in infancy and may manifest as hematopoietic failure, increased intracranial pressure, and neurological dysfunction. Without timely intervention, most affected individuals die within the first few years of life.^[3]^ Mutations in the TCIRG1 gene lead to ARO by disrupting the function of the a3 subunit of the V-ATPase proton pump, resulting in impaired acidification by osteoclasts and defective bone resorption.^[4]^

## 2. Methods

This study was conducted in accordance with the ethical and scientific principles of the Declaration of Helsinki and complied with Chinese regulations for Good Clinical Practice. Ethical approval was obtained from the Ethics Committee of The First People’s Hospital of Datong, Shanxi, China (approval number: [2025] 01). Written informed consent for publication of clinical details and images was obtained from the patient’s guardian. Clinical data of the patient were obtained from hospital medical records and direct clinical observation. The diagnostic work-up included physical examination, laboratory testing (complete blood count, C-reactive protein), and imaging studies (plain radiographs and computed tomography). Radiographic findings were assessed for characteristic features of osteopetrosis, including bone-in-bone appearance and sandwich vertebrae. Genetic testing was performed using next-generation sequencing, and identified compound heterozygous mutations in the *TCIRG1* gene.

## 3. Case report

The patient was a female infant, aged 6 months and 16 days at admission, presenting with intermittent high fever for 4 days. She had been diagnosed with osteopetrosis at 3 months of age and had no known family history. Physical examination revealed a bulging anterior fontanel, hepatosplenomegaly, and lethargy. Laboratory findings were as follows: hemoglobin 94 g/L, platelet count 52 × 10⁹/L, and C-reactive protein 11.9 mg/L.

**Figure 1.**
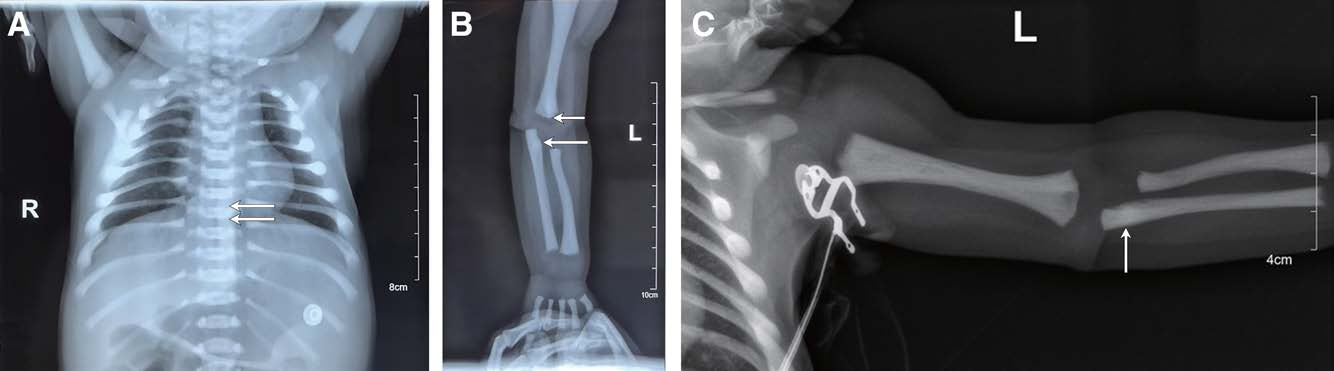
(A) Thoracic spine X-ray showing sandwich vertebrae with sclerotic endplates and a central radiolucent band (arrows). (B) Radiograph of the left arm showing bone-in-bone appearance at the distal humerus and proximal ulna (arrows). (C) Lateral view of the left arm showing metaphyseal sclerosis with bone-in-bone appearance at the proximal ulna (arrow).

Imaging studies revealed sandwich vertebrae in the thoracic spine (Fig. 1A), characterized by sclerotic endplates and a central radiolucent band; bone-in-bone appearance in the diaphyses of long bones (Fig. 1B and C).

Genetic testing identified compound heterozygous mutations in the *TCIRG1* gene: c.1216G >T (inherited from the father) and c.1384_1386del (inherited from the mother), consistent with a diagnosis of ARO type 1 (ARO1, OMIM #259700).

Supportive and anti-infective treatments were administered. However, hematopoietic stem cell transplantation (HSCT) was not performed due to the guardians’ decision. The patient was discharged and later died from severe bone marrow suppression and infection. Additional details of this case are presented in Table 1.

**Table 1:** Key clinical timeline with diagnosis and management.

Date	Event/key findings/examinations	Diagnosis and management
August 24, 2021	Birth: vaginal delivery, GA 40 wk	NA
September 16, 2021	PLT persistently low (96 × 10^9^/L), measured at the local facility	Referral to a higher-level hospital was recommended
October 21, 2021	Admitted to Children’s Hospital of Shanxi: pale complexion, petechiae, splenomegaly. Hb 81 g/L, PLT 47 × 10^9^/L. Abdominal US: splenomegaly, ascites.	Dx: anemia, splenomegaly (etiology unknown). Symptomatic hemostasis.
October 23, 2021	Imaging examination (1): chest X-ray demonstrated “sandwich vertebrae.” Bone marrow biopsy: hypoplasia, absence of megakaryocytes.	Case discussion: suspected osteopetrosis, genetic testing recommended.
October 24, 2021	Imaging examination (2): plain radiographs of the long bones revealed increased bone mineral density accompanied by widening of the metaphyses, “bone-in-bone” appearance.	Supportive of osteopetrosis diagnosis.
October 25, 2021	Discharge from Children’s Hospital of Shanxi	Diagnosis of osteopetrosis
January 18, 2022	Outpatient follow-up: Abdominal US: splenomegaly	Instructions: follow-up observation
March 8, 2022	Outpatient follow-up: WBC 15.79 × 10^9^/L, Hb 94 g/L, PLT 52 × 10^9^/L	Anemia, thrombocytopenia
March 9, 2022	Genetic testing: TCIRG1 mutation confirmed	ARO type I confirmed
March 12, 2022	Admitted to our hospital: 4-d fever (max 40℃), bulging fontanel, hepatosplenomegaly. PLT 48 × 10^9^/L.	DX: fever (unclear cause), osteopetrosis, increased ICP, mild anemia, thrombocytopenia, splenomegaly. Tx: ceftriaxone (anti-infection), mannitol (ICP lowering), IV fluids.
March 13, 2022	Disease deterioration: recurrent fever, vomiting, diarrhea	Viral tests negative. Supportive therapy continued.
March 14, 2022	Clinical Improvement: afebrile, improved mental status and feeding	Continued previous treatment
March 15, 2022	Symptom recurrence: fever (38.7℃), vomiting × 4, watery stools	Mannitol discontinued. Continued antibiotics and supportive care.
March 16, 2022	Discharge from our hospital Stable, no fever, no seizures.	Discharge Dx: acute pharyngitis, osteopetrosis, possible increased ICP, anemia, thrombocytopenia, splenomegaly, transient hypogammaglobulinemia of infancy, rickets. Discharge advice: VitAD, oral calcium, prednisolone 5mg bid × 1Wk.
August 24, 2022	Disease progression: Wt 7.5 kg, macrocephaly (AF 5 × 5 cm), can sit but cannot stand.	Growth and developmental delay. Parental refusal of further treatment optioned for home palliative care.
August 23, 2023	Slow progression: intermittent petechiae, no weight gain, progressive macrocephaly, scalp veins prominent, sunset eyes, hearing loss, 8 deciduous teeth crumbling.	Supportive/palliative care at home.
January 27, 2024	Symptom recurrence: cough, increased petechiae, marked abdominal distension, spleen below umbilicus.	NA
February 22, 2024	Disease worsening: poor responsiveness, poor feeding, groaning, night crying, progressive abdominal distension, prominent abdominal wall veins.	Parents refused investigations and treatment.
March 8, 2024	Death at home	–

Abdominal US = abdominal ultrasound, AF = anterior fontanelle, ARO = autosomal recessive osteopetrosis, bid = bis in die (twice a day), Dx = diagnosis, GA = gestational age, Hb = hemoglobin, ICP = intracranial pressure, IV = intravenous, PLT = platelet count, Tx = treatment, Wk = week, Wt = weight.

## 4. Discussion

The clinical manifestations of osteopetrosis arise from 2 primary pathophysiological processes: first, bone marrow failure due to narrowed medullary cavities, resulting in anemia, immune dysfunction, and bleeding tendency; and second, structural abnormalities caused by increased bone density and impaired bone remodeling, leading to progressive compression of the nervous system.^[1,4,5]^ In this case, the patient’s progressive anemia, thrombocytopenia, and hepatosplenomegaly were likely related to the replacement of marrow space by abnormal bone tissue, resulting in compensatory extramedullary hematopoiesis. The presence of increased intracranial pressure and bilateral positive Babinski signs in this patient suggests involvement of the central nervous system.

In patients with ARO, impaired bone remodeling leads to increased bone density, particularly in the skull base. This process results in narrowing of the cranial nerve foramina, which can compress the cranial nerves passing through them, including the optic nerve. Optic nerve compression is one of the most common neurological complications in ARO and typically presents as visual impairment or vision loss.^[4,5]^ Allogeneic HSCT is an effective treatment for ARO.^[2,6]^ Genetic counseling is essential for families affected by osteopetrosis. Genetic testing not only aids in confirming the diagnosis but also facilitates prenatal diagnosis and family screening.^[3,7]^ For families with a known history of the disease, preconception genetic counseling and genetic screening are recommended to reduce the risk of disease occurrence.^[1]^

This case offers a classic example of how timely recognition of radiographic hallmarks, such as sandwich vertebrae and bone-in-bone appearance, can facilitate early diagnosis of ARO. It underscores the indispensable role of genetic testing in confirming the diagnosis and guiding family counseling. In this case, HSCT was not performed, and the patient eventually died of marrow failure and infection. While supportive treatment may offer temporary relief, current evidence suggests that HSCT remains the only potentially curative option for ARO. Therefore, early evaluation of transplant eligibility and timely referral should be considered whenever feasible. This case also highlights the need to raise awareness of rare bone disorders in clinical practice to avoid delayed diagnosis and missed opportunities for intervention.

This case report has several limitations. First, as a single case report, the findings cannot be generalized to the wider population. Second, HSCT was not performed in this patient, which limited our ability to evaluate therapeutic efficacy. Third, follow-up data on imaging and genetic testing were limited, restricting further assessment of disease progression.

## 5. Conclusion

This case illustrates the classic clinical course of ARO in infancy and underscores the importance of early recognition. Plain radiographs remain valuable for the initial suspicion of the disease, while genetic testing is indispensable for definitive diagnosis and family counseling. Although supportive therapy may provide temporary relief, HSCT is currently the only curative option. Timely evaluation of transplant eligibility and prompt referral are therefore essential to improve outcomes in affected infants.

## Acknowledgments

All authors extend their sincere gratitude to Bothwin Clinical Study Consultant for their invaluable support in figure preparation.

## Author contributions

**Conceptualization:** Fang Hou.

**Data curation:** Fang Hou.

**Investigation:** Fang Hou.

**Visualization:** Junyan Zhang.

**Writing – original draft:** Junyan Zhang.

**Writing – review & editing:** Fang Hou, Junyan Zhang.
